# Effects of Music Therapy and Inhalation Aromatherapy on Pain Intensity, Anxiety, and Fear Levels in Patients Undergoing Coronary Angiography: A Randomized Controlled Trial

**DOI:** 10.1155/prm/9447650

**Published:** 2026-02-17

**Authors:** Fatma Gür, Gülcan Bahcecioglu Turan

**Affiliations:** ^1^ Department of Internal Medicine Nursing, Institute of Health Sciences, Fırat University, Elazığ, Turkey, firat.edu.tr; ^2^ Department of Nursing, Faculty of Health Sciences, Fırat University, Elazığ, Turkey, firat.edu.tr

**Keywords:** anxiety, aromatherapy, coronary angiography, music therapy, pain

## Abstract

**Objective:**

To examine the effects of music therapy, inhalation aromatherapy, and their combination on pain intensity, anxiety, and fear in patients undergoing coronary angiography.

**Methods:**

This randomized controlled trial included 128 patients assigned to four groups: control, music therapy, aromatherapy, and combined music + aromatherapy. Interventions were applied 5–10 min before the procedure and continued during angiography. Visual analog scales for assessing pain (VAS‐P), anxiety (VAS‐A), and fear (VAS‐F), and the State Anxiety Inventory (SAI) were assessed pre‐ and postprocedure. Data were analyzed using mixed‐design ANOVA to examine time, group, and time × group effects, and effect sizes (*η* ^2^) were calculated.

**Results:**

Significant time × group interactions were found for pain (*η* ^2^ = 0.205), fear (*η* ^2^ = 0.527), anxiety (*η* ^2^ = 0.550), and state anxiety (*η* ^2^ = 0.546) (all *p* < 0.001). All intervention groups showed greater reductions in pain, anxiety, and fear compared with the control group (*p* < 0.001). No significant differences were observed among the three intervention groups (*p* > 0.05), although the combined intervention yielded the largest improvements.

**Conclusion:**

Music therapy and inhalation aromatherapy reduce pain, anxiety, and fear in patients undergoing coronary angiography effectively and can be recommended as nonpharmacological nursing interventions.

**Trial Registration:** ClinicalTrials.gov.: NCT05622383

## 1. Introduction

Cardiovascular diseases (CVDs) remain the leading cause of death globally, accounting for approximately 19.2 million deaths in 2023 (American College of Cardiology [ACC]) [1]. The global prevalence of CVD has exceeded 626 million people, with a substantial rate attributable to modifiable risk factors such as hypertension, unhealthy diet, and environmental exposures [2]. Given this epidemiological burden, coronary angiography (CAG) has become an essential diagnostic and interventional procedure in contemporary cardiology, enabling timely detection and management of coronary artery disease (CAD) [3]. CAG is an invasive procedure in which a radiopaque contrast agent is introduced via catheterization—usually through the radial or femoral artery—to visualize the coronary arteries. It provides detailed information on arterial stenoses and occlusions, making it the “gold standard” for diagnosing CAD and guiding revascularization strategies [4]. Despite its clinical value, patients often experience pain, anxiety, and fear because they remain conscious during the procedure, and hemodynamic fluctuations can increase the risk of vascular access complications [5–8].

To mitigate these negative experiences, nonpharmacological interventions have gained attention in nursing practice. Interventions such as music therapy, massage, and other complementary strategies are minimally invasive, low‐risk, cost‐effective, and feasible to implement within nursing care, serving as alternatives or complements to pharmacological methods [9].

Aromatherapy, the use of plant‐derived essential oils, has demonstrated effectiveness in reducing stress, anxiety, and pain in clinical settings. Inhalation of lavender oil provides sedative and analgesic effects through its active components, linalool and linalyl acetate, which modulate neurochemical pathways [10, 11]. Aromatherapy aligns with holistic nursing principles, enabling nurses to enhance patient comfort, support psychological well‐being, and deliver individualized care [12].

Similarly, music therapy is a noninvasive nursing intervention that distracts attention, reduces fear, and improves procedural experience. Music modulates the autonomic nervous system, enhances endorphin secretion, and provides emotional comfort [13]. Its low cost, ease of implementation, and minimal risk make it suitable for integration into routine nursing care, promoting patient‐centered, holistic interventions.

Although prior studies have examined the effects of aromatherapy or music therapy individually during CAG, evidence regarding their combined application remains limited [14–16]. Existing studies have predominantly focused on single interventions or isolated outcomes, and few have evaluated the complementary or potential synergistic effects of these nonpharmacological approaches within the same clinical setting. In addition, direct comparisons of these interventions under a standardized nursing care protocol are scarce. Therefore, this study aims to address this knowledge gap by investigating the combined effects of music therapy and inhalation‐based aromatherapy on pain, anxiety, and fear levels in patients undergoing CAG. By evaluating these interventions as holistic nursing practices implemented concurrently during the procedure, this trial provides novel evidence regarding their complementary role in enhancing patient comfort and supporting hemodynamic stability during invasive cardiac procedures.

### 1.1. Research Hypotheses

 H0: Music therapy and inhalation aromatherapy do not affect pain intensity, anxiety, and fear levels in patients who are scheduled for CAG.
 H1: Music therapy and inhalation aromatherapy affect pain intensity, anxiety, and fear levels in patients who are scheduled for CAG.

## 2. Methods

### 2.1. Trial Design

This research utilized a randomized, pretest–posttest design with control and intervention groups to evaluate the effects of the interventions.

### 2.2. Participants and Setting

**FIGURE 1:**
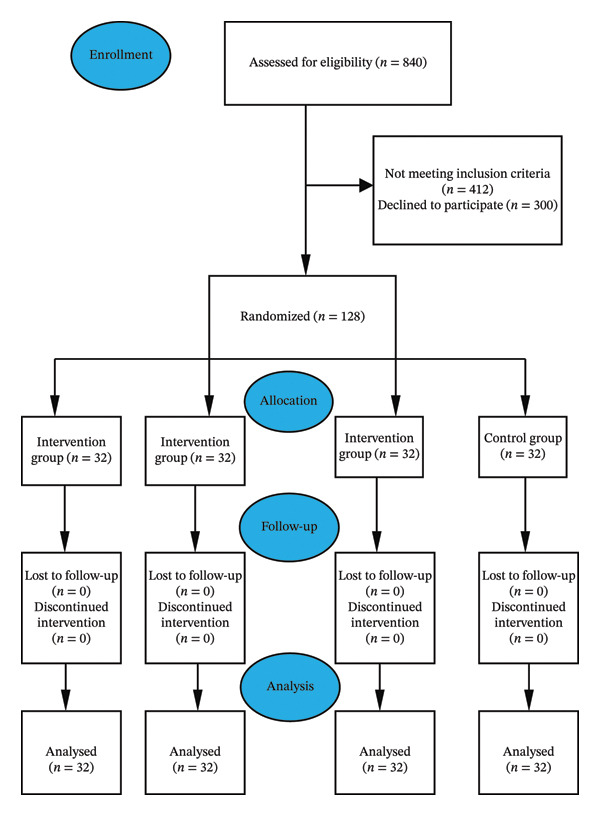
CONSORT diagram.

The population for this study included individuals who received CAG procedures at two medical centers (a university hospital and a state hospital) in eastern Türkiye from November 2022 to June 2023. The sample of the study comprised 128 CAG patients, including 96 in the intervention group (1: music, 2: aroma, and 3: music + aroma) and 32 in the control group, who agreed to take part in the study and who were randomly selected from the defined population that met the research criteria. Inclusion criteria consisted of individuals aged ≥ 18 years, free from cognitive and communicative impairments, and not under sedative use before or during the procedure. Exclusion criteria were having an emergency CAG, having an obstacle to smell, and having a known allergy to the essential oils to be applied. The necessary sample size was calculated in advance using the G‐Power 3.1.9.4 software for power analysis. As a reference from the studies on the topic, the effect size was found to be 0.30 by considering the mean postprocedure visual analog scale for anxiety (VAS‐A) score of the music, aroma, and control groups in Lee et al.’s study [17]. In line with these results, it was found according to the one‐way analysis of variance (ANOVA) test in the Power Analysis Program that there should be a total of 124 patients, with a minimum of 31 in each group, with an effect size of 0.30, a significance level of 0.05 [18], and a power of 0.80 [19]. Out of 840 patients assessed for eligibility, the study was finalized with 128 participants (32 per group: music, aroma, music + aroma, and control), following the exclusion of 412 candidates for not conforming to the inclusion criteria and 300 who declined participation (Figure 1).

### 2.3. Randomization

Randomization was performed using the “Random Integer Generator” tool on https://Random.org, generating a single column of numbers from 1 to 128, with each of the numbers 1, 2, 3, and 4 appearing an equal number of times. Participants meeting the study inclusion criteria were randomly assigned to these numbers. Group identities (1 = control, 2 = music, 3 = aromatherapy, and 4 = music + aromatherapy) were determined at the start of the study by an independent researcher through a lot‐drawing procedure, and the assignments were kept in sealed opaque envelopes to ensure allocation concealment. Although blinding was not implemented for participants or the primary investigator due to the nature of the interventions, the statistician responsible for data analysis was blinded to the group assignments. This approach helped reduce potential bias during the interpretation of results. The study adhered to CONSORT (2017) guidelines throughout (see Figure 1).

### 2.4. Measurements

Data collection instruments included the patient information form, the visual analog scale for pain (VAS‐P), VAS‐A, visual analog scale for fear (VAS‐F), and the State Anxiety Inventory (SAI), all of which were administered through face‐to‐face interviews.

#### 2.4.1. Patient Information Form

This form was developed by the researcher based on the existing literature [20, 21] to gather demographic and health‐related information. It consisted of 11 items addressing variables such as chronic disease status, gender, place of residence, age, educational background, marital status, income level, smoking habits, and previous experience with CAG.

#### 2.4.2. VAS‐P

VAS‐P was used to evaluate subjective experience of unidimensional pain [22] in adults and was found to be reliable and valid. Previous studies reported it to have higher sensitivity than other methods. It is a scale in the form of a 0–10‐cm ruler to evaluate pain quantitatively (0: *no pain*, 10: *highest pain severity*) [23, 24].

#### 2.4.3. VAS‐F

A 10‐cm VAS‐F, oriented either vertically or horizontally, was used for patients to self‐report their fear levels in both resting and activity states. The scale is numbered from 0 to 10, with 0 indicating no fear and 10 indicating the most intense fear. Although no formally validated Turkish version of the VAS‐F is currently available, visual analog scales are supported by methodological literature as practical and sensitive tools for assessing subjective and momentary emotional states such as fear and preprocedural anxiety in clinical settings [25–27]. In the present study, the scale was translated and back‐translated by bilingual experts and pilot‐tested to ensure linguistic clarity and patient comprehensibility; however, no formal psychometric validation was performed.

#### 2.4.4. VAS‐A

VAS‐A consists of a single item designed to assess anxiety intensity on a 10‐cm horizontal line ranging from 0 (*not anxious*) to 10 (*very anxious*). Higher scores indicate higher levels of perceived anxiety. The VAS‐A was used to evaluate participants’ immediate and subjective perception of anxiety before and after the procedure, allowing for the detection of short‐term changes in anxiety levels [21, 24].

#### 2.4.5. SAI

The Turkish adaptation and psychometric evaluation of the SAI were conducted by Öner and Le Compte (1983), based on the original scale developed by Spielberger [28]. In the present study, the 20‐item SAI was used to assess state anxiety as a multidimensional construct, reflecting individuals’ cognitive and emotional responses to specific conditions. The scale is structured as a 4‐point Likert‐type instrument, yielding total scores ranging from 20 to 80, with higher scores indicating greater anxiety levels. The original validation studies reported high internal consistency, with Cronbach’s alpha values between 0.94 and 0.96. In the present study, internal consistency was also satisfactory, with Cronbach’s alpha values ranging from 0.80 to 0.941 across pre‐ and postintervention assessments [29].

### 2.5. Rationale for the Concurrent Use of VAS‐A and SAI

The concurrent use of the VAS‐A and the SAI was intended to capture complementary dimensions of anxiety. The VAS‐A provides a rapid and sensitive assessment of immediate, subjective anxiety intensity, whereas the SAI offers a standardized and theoretically grounded evaluation of state anxiety across multiple emotional and cognitive domains. Using both instruments enabled a more comprehensive assessment of anxiety beyond what could be achieved with a single measure.

### 2.6. Control Group

In the control group, patients completed the patient information form along with the VAS‐P, VAS‐F, VAS‐A, and SAI scales approximately 30 min before CAG to establish baseline values. Throughout the procedure, no additional interventions were implemented; participants received only standard clinical care. Fifteen minutes after the procedure, the same scales were reapplied to assess any postprocedural changes.

### 2.7. Intervention Groups

Intervention group participants were assigned to one of the three groups: music therapy, aromatherapy, or a combination of music therapy and aromatherapy. Baseline measurements using the patient information form, VAS‐P, VAS‐F, VAS‐A, and SAI were conducted 30 min before CAG. Postprocedure outcomes were assessed 15 min after CAG using the same instruments.

The durations of the interventions were determined in accordance with previous literature suggesting that short‐term applications (5–15 min) of music and aromatherapy are sufficient to promote relaxation and reduce preprocedural anxiety and stress [10, 14, 17]. Therefore, music therapy was applied for 10 min and aromatherapy for 5 min, in line with these findings and expert recommendations.

1. Music therapy group: The Group for the Research and Promotion of Turkish Music was contacted for the preparation of the music used in this group. In line with the recommendations of this group, a music recording was prepared based on the maqams traditionally used in music therapy practices, both in the past and today. Patients listened to this recording via Bluetooth and over‐ear headphones for 10 min in the waiting room and continuously throughout the CAG procedure. Each set of over‐ear headphones was disinfected with alcohol‐based antiseptic wipes before and after use to maintain infection control standards.
2. Aromatherapy group: For this group, pure (100% undiluted) lavender essential oil was used for aromatherapy. The oil was supplied by Nativital Natural Life and Health Products Co. (Türkiye), a manufacturer operating under the Turkish Cosmetic Law (Law No. 5324, Article 7, dated March 24, 2005). The selection of the oil was made in consultation with Dr. Melike Özberk Koç (pediatrician and phytotherapist) after a review of the relevant literature [10]. Lavender oil was preferred because of its analgesic, calming, and soothing effects, which help reduce anxiety and stress associated with pain [11, 30]. Two drops of the oil were applied to a piece of sterile gauze, which was then placed on the patient’s shoulder area to allow inhalation. Inhalation, chosen as a simple and effective method that yields rapid results [12], was performed for 5 min in the waiting room and continued throughout CAG.
3. Music therapy + aromatherapy group: Patients in this group simultaneously listened to the prepared music recording for 10 min in the waiting room and inhaled lavender oil for 5 min. Both interventions were maintained continuously during the CAG procedure.

After a 15‐min recovery period post‐CAG, patients were taken to their beds in the ward, and second evaluations were performed using VAS‐P, VAS‐F, VAS‐A, and SAI.

### 2.8. Evaluation of Data

Data analysis was conducted using IBM SPSS Statistics Version 26 (IBM Corp., Armonk, NY, USA). Descriptive statistics were presented as frequencies (*n*), percentages (%), means and standard deviations (mean ± SD), medians (M), and minimum and maximum values. Normality of numerical variables was assessed using the Shapiro–Wilk test. For comparisons among groups at a single time point, one‐way ANOVA was performed for numerical variables. To evaluate changes across multiple time points and between groups, mixed‐design ANOVA was conducted. Sphericity was assessed using Mauchly’s test, and the Greenhouse–Geisser correction was applied when sphericity was violated. Post hoc comparisons for significant main and interaction effects were performed using the Bonferroni test. Results are presented as mean ± SD and 95% confidence intervals (CIs), with *p* < 0.05 considered statistically significant. Categorical variables were analyzed using Pearson’s chi‐square test or Fisher’s exact test, as appropriate.

## 3. Results

Table 1 shows that the patients in the control, music therapy, aroma, and music + aroma groups were similar regarding gender, marital status, residence, education, employment, income, smoking status, chronic disease, previous CAG, reason for CAG, and age (*p* > 0.05), indicating a homogeneous distribution across groups.

**TABLE 1:** Comparison of groups according to their descriptive characteristics (*n* = 128).

	**Groups**				**Test statistics**	
	**Control** ***n = *32**	**Music** ***n = *32**	**Aroma** ***n = *32**	**Music + Aroma** ***n = *32**	**Test value**	**p**
*Age*						
*Mean ± SD*	69.44 ± 9.37	69.53 ± 12.62	66.53 ± 11.48	67.38 ± 10.49	0.591^‡^	0.622
*M* (*min–max*)	69 (50–90)	69 (49–90)	69 (47–90)	69 (40–87)		

*Gender*						
Female	15 (46.9%)	14 (43.8%)	15 (46.9%)	15 (46.9%)	0.094^†^	0.993
Male	17 (53.1%)	18 (56.3%)	17 (53.1%)	17 (53.1%)		

*Marital Status*						
Married	28 (87.5%)	27 (84.4%)	27 (84.4%)	26 (81.3%)	0.474^†^	0.925
Single	4 (12.5%)	5 (15.6%)	5 (15.6%)	6 (18.8%)		

*Place of residence*						
City	21 (65.6%)	22 (68.8%)	22 (68.8%)	23 (71.9%)	0.853^†^	0.991
Town	9 (28.1%)	8 (25%)	9 (28.1%)	7 (21.9%)		
Village	2 (6.3%)	2 (6.3%)	1 (3.1%)	2 (6.3%)		

*Educational status*						
Literate	8 (25.0%)	7 (21.%9)	9 (28.1%)	3 (9.4%)		
Elementary	18 (56.3%)	20 (62.5%)	17 (53.1%)	19 (59.4%)	12.344^†^	0.418
Secondary	4 (12.5%)	4 (12.5%)	2 (6.3%)	7 (21.%9)		
High school and above	2 (6.3%)	1 (3.1%)	4 (12.5%)	3 (9.3%)		

*Employment status*						
Employed	4 (12.5%)	6 (18.8%)	7 (21.9%)	2 (6.3%)	3.647^†^	0.302
Unemployed	28 (87.5%)	26 (81.3%)	25 (78.1%)	30 (93.2%)		

*Income status*						
Income < expense	6 (18.8%)	4 (12.5%)	3 (9.4%)	4 (12.5%)	5.155	0.524
Income = expense	25 (78.1%)	26 (81.3%)	29 (90.6%)	28 (87.5%)		
Income > expense	1 (3.1%)	2 (6.3%)	—	—		

*Smoking status*						
Yes	14 (43.8%)	17 (53.1%)	15 (46.9%)	17 (53.1%)	0.844^†^	0.839
No	18 (56.3%)	15 (46.9%)	17 (53.1%)	15 (46.9%)		

*Presence of chronic disease*						
Yes	23 (71.9%)	24 (75%)	21 (65.%6)	24 (75%)	0.928^†^	0.819
No	9 (28.1%)	8 (25%)	11 (34.4%)	8 (25%)		

*Chronic angiography*						
Yes	12 (37.5%)	10 (31.2%)	10 (31.3%)	13 (40.6%)	0.925^†^	0.819
No	20 (62.5%)	22 (68.8%)	22 (68.8%)	19 (50.9%)		

*Note:* Summary statistics are given as *mean ± standard deviation* and *median* (*minimum, maximum*) for numerical data and *number* (*percentage*) for categorical data.

^‡^ANOVA (*F*).

^†^Chi‐square test (*χ* ^2^).

Preprocedure pain scores did not differ significantly among groups (*p* > 0.05). Postprocedure scores decreased significantly in all intervention groups compared to the control group, with the largest reduction observed in the aroma group. Post hoc Bonferroni analysis (Table 2) confirmed that the control group’s postprocedure pain scores were significantly higher than those of the intervention groups, while no differences were detected among the intervention groups themselves. Intragroup analyses showed significant reductions in pain scores in the intervention groups (*p* < 0.05) but not in the control group (Table 2).

**TABLE 2:** Comparison of mean intragroup and intergroup pain and fear scores of patients.

	**Groups**				**Intergroup test statistics**		
	**Control** ***n = *32**	**Music** ***n = *32**	**Aroma** ***n = *32**	**Music + Aroma** ***n = *32**	**F**	**p**	**η** ^2^
	**X ± SS**	**X ± SS**	**X ± SS**	**X ± SS**			
*Pain*							
Preprocedure	3.06 ± 0.67^*a*^	2.84 ± 0.68^*a*^	2.91 ± 0.73^*a*^	3.22 ± 0.66^*a*^	1.923	0.129	0.044
Postprocedure	2.88 ± 0.83^*a*^	2.00 ± 0.88^*b*^	1.81 ± 0.74^*b*^	2.22 ± 0.75^*b*^	**10.657**	**< 0.001**	**0.205**
Intragroup test statistics	*F = *2.592 *p* = 0.110 *η* ^2^ = 0.020	***F = *52.495** **p** < 0.001 **η** ^2^ = 0.297	***F = *88.211** **p** < 0.001 **η** ^2^ = 0.416	***F = *73.738** **p** < 0.001 **η** ^2^ = 0.373			
Difference (pre–post procedure)	−0.19 ± 0.47	−0.84 ± 0.88	−1.09 ± 0.53	−1.00 ± 0.67	**12.338**	**< 0.001**	**0.230**

*Fear*							
Preprocedure	6.81 ± 1.33^*a*^	6.84 ± 1.05^*a*^	6.56 ± 1.11^*a*^	6.78 ± 0.91^*a*^	0.424	0.736	0.010
Postprocedure	5.63 ± 1.26^*b*^	3.28 ± 0.96^*c*^	2.97 ± 1.00^*c*^	2.97 ± 1.03^*c*^	**46.137**	**< 0.001**	**0.527**
Intragroup test statistics	***F = *43.940** **p** < 0.001 **η** ^2^ = 0.262	***F = *395.461** **p** < 0.001 **η** ^2^ = 0.761	***F = *402.429** **p** < 0.001 **η** ^2^ = 0.764	***F = *452.912** **p** < 0.001 **η** ^2^ = 0.785			
Difference (pre–post procedure)	−1.19 ± 0.78	−3.56 ± 1.13	−3.59 ± 1.01	−3.81 ± 1.09	**47.863**	**< 0.001**	**0.537**

*Note:* Mixed design ANOVA (*F*), effect size (**η** ^2^), X ± SD = *mean ± standard deviation*. Bold parts are statistically significant (*p* < 0.05). *a > b > c*: different letters or letter combinations in the same row indicate statistically significant differences (*p* < 0.05).

Preprocedure fear scores were similar across groups (*p* > 0.05). Postprocedure fear scores decreased significantly in all groups, with the largest reduction in the music + aroma group. Post hoc analysis (Table 2) indicated higher scores in the control group compared to the intervention groups, while no significant differences were found among the intervention groups. Intragroup analyses confirmed significant reductions in fear scores across all groups (*p* < 0.05) (Table 2).

All groups showed significant decreases in anxiety and state anxiety scores following the procedure (*p* < 0.05), with the greatest reductions observed in the music + aroma group. Preprocedure scores did not differ among groups (*p* > 0.05), whereas postprocedure assessments revealed significant differences (*p* < 0.05). Post hoc analysis (Table 3) confirmed that the control group had higher postprocedure anxiety and state anxiety scores than the intervention groups, with no differences among the intervention groups (Table 3).

**TABLE 3:** Comparison of intragroup and intergroup mean anxiety and state anxiety scores of patients.

	**Groups**				**Intergroup test statistics**		
	**Control** ***n = *32**	**Music** ***n = *32**	**Aroma** ***n = *32**	**Music + Aroma** ***n = *32**	***F***	**p**	**η** ^2^
	**X ± SD**	**X ± SD**	**X ± SD**	**X ± SD**			
*Anxiety*							
Preprocedure	6.66 ± 1.18^*a*^	6.72 ± 1.11^*a*^	6.59 ± 1.48^*a*^	7.09 ± 0.89^*a*^	1.150	0.332	0.027
Postprocedure	5.38 ± 1.24^*b*^	3.38 ± 0.91^*c*^	2.63 ± 0.91^*c*^	3.06 ± 0.76^*c*^	**50.468**	**< 0.001**	**0.550**
Intragroup test statistics	***F = *54.452** **p** < 0.001 **η** ^2^ = 0.305	***F = *370.866** **p** < 0.001 **η** ^2^ = 0.749	***F = *522.465** **p** < 0.001 **η** ^2^ = 0.808	***F = *539.05** **p** < 0.001 **η** ^2^ = 0.813			
Difference (pre–post procedure)	−1.28 ± 0.77	−3.34 ± 1.04	−3.97 ± 1.18	−4.03 ± 0.90	**55.025**	**< 0.001**	**0.571**

*State Anxiety*							
Preprocedure	53.56 ± 7.81^*a*^	50.47 ± 4.84^*a*^	52.28 ± 6.90^*a*^	53.75 ± 4.19^*a*^	1.957	0.124	0.045
Postprocedure	45.09 ± 7.15^*b*^	31.41 ± 4.37^*c*^	32.34 ± 5.45^*c*^	31.84 ± 3.62^*c*^	**49.714**	**< 0.001**	**0.546**
Intragroup test statistics	***F = *79.217** **p** < 0.001 **η** ^2^ = 0.390	***F = *401.367** **p** < 0.001 **η** ^2^ = 0.764	***F = *439.06** **p** < 0.001 **η** ^2^ = 0.780	***F = *530.052** **p** < 0.001 **η** ^2^ = 0.810			
Difference (pre–post procedure)	−8.47 ± 4.08	−19.06 ± 5.90	−19.94 ± 5.75	−21.91 ± 5.60	**40.229**	**< 0.001**	**0.493**

*Note:* Mixed design ANOVA (*F*), effect size (**η** ^2^), X ± SD = *mean ± standard deviation*. Bold parts are statistically significant (*p* < 0.05). *a > b > c*: different letters or letter combinations in the same row indicate statistically significant differences (*p* < 0.05).

## 4. Discussion

Interventions are necessary parts of diagnosis and treatment processes but often evoke anxiety and fear in patients. Psychological factors can influence the perception and intensity of pain in various ways [31]. Remaining conscious throughout the CAG procedure may further heighten anxiety due to pain anticipation, potential risks, and uncertainty about the results [32]. This study aimed to evaluate the effects of music therapy and inhalation‐based aromatherapy on pain, anxiety, and fear in patients undergoing CAG.

The absence of significant differences in baseline characteristics among the control and intervention groups confirmed their demographic and clinical homogeneity. Postprocedural analyses revealed significantly lower pain scores in all intervention groups compared to the control group, though no significant differences were found among the intervention groups. The largest reduction was observed in the aromatherapy group. This finding aligns with previous research indicating that both music and aromatherapy effectively reduce perceived pain in clinical populations [33–39]. Music is known to influence the limbic system and autonomic functions, triggering endorphin and enkephalin release, which modulate pain perception and emotional state [35, 40]. Similarly, aromatherapy acts via olfactory–limbic pathways; volatile compounds in essential oils can stimulate the release of neurotransmitters such as dopamine and serotonin, leading to analgesic and relaxing effects [41]. The combined use of music and aromatherapy may provide additive effects through their mutual modulation of the limbic system [41–43]. These results support the use of music and/or aromatherapy as nonpharmacological nursing interventions for pain management during CAG.

Fear levels also decreased significantly in the intervention groups compared to the control group, with the greatest reduction observed in the music therapy + aromatherapy group. This improvement may be attributed to the calming effects of both interventions, while the reduction observed in the control group likely resulted from the relief experienced at the end of the procedure. Prior studies have demonstrated the fear‐reducing effects of both music and aromatherapy, likely mediated through neuroendocrine regulation and increased levels of serotonin and dopamine [44–49]. The combined use of music and aromatherapy further amplifies these effects, promoting relaxation and reducing emotional distress during invasive procedures [14, 16].

Similarly, both music and aromatherapy interventions led to significant reductions in anxiety and state anxiety levels compared to the control group. The greatest decrease was again noted in the combined intervention group. These results are consistent with previous studies showing that both interventions reduce preprocedural anxiety by eliciting relaxation responses through limbic and hypothalamic activation [50–53]. The synergistic effect observed when combining music and aromatherapy suggests that these complementary modalities enhance one another’s anxiolytic effects by promoting relaxation, distraction, and emotional regulation [14–16, 52]. Therefore, music therapy and/or aromatherapy can be recommended as effective, noninvasive nursing strategies for reducing anxiety and fear in patients undergoing CAG.

Overall, this study contributes to the growing body of evidence supporting the integration of music and aromatherapy as holistic nursing interventions in invasive cardiac procedures, helping to improve patient comfort and psychological well‐being.

## 5. Limitations and Strengths of the Study

One major limitation of this study is its confinement to two research sites, which may limit the generalizability of the findings to other populations or healthcare settings. The sample size, determined by the minimum statistical power requirement, also limits generalizability. Patient preferences for the type of music or essential oil were not considered, representing another limitation. Additionally, outcomes were assessed only 15‐min postprocedure, which may not reflect the sustained or long‐term effects of the interventions. Two instruments (VAS‐A and SAI) were used to assess anxiety; while this provided a comprehensive evaluation of both immediate subjective anxiety (VAS‐A) and broader state anxiety (SAI), it may have introduced a degree of overlap between the measures.

Furthermore, angiographic outcomes (e.g., normal results, stent placement, or referral for surgery) and the specific access technique used (radial, femoral, or brachial artery) were not recorded, which could influence patients’ psychological and pain responses. Finally, the study was terminated early upon reaching the minimum sample size due to space and time limitations.

Despite these limitations, the inclusion of multiple intervention groups and the use of validated assessment instruments represent notable methodological strengths of the study.

In addition, fear was assessed using the VAS‐F, which has not been formally validated in the specific population undergoing CAG. Although visual analog scales are widely used in clinical research to assess subjective and momentary emotional states such as pain, anxiety, and fear, the lack of population‐specific psychometric validation should be considered a methodological limitation. Nevertheless, VAS‐based instruments are regarded as practical and sensitive tools in acute clinical settings, particularly when rapid assessment is required. Therefore, fear‐related findings should be interpreted with caution, and future studies are recommended to employ fully validated fear‐specific instruments or conduct validation studies of VAS‐F in similar patient populations.

## 6. Conclusion

It was found that music therapy and inhalation aromatherapy applied to patients undergoing CAG decreased pain intensity, anxiety, and fear levels of patients. Based on these results, implementing music therapy sessions and/or inhalation aromatherapy may serve as a beneficial nursing practice for alleviating pain, anxiety, and fear in patients scheduled to undergo CAG.

## Author Contributions

Gülcan Bahcecioglu Turan: conceptualization, methodology, investigation, writing–original draft, writing–review and editing. Gülcan Bahcecioglu Turan: conceptualization, methodology, investigation, and writing–original draft. Fatma Gür: methodology, writing–original draft, and writing–review and editing.

## Funding

This research did not receive any specific grant from funding agencies in the public, commercial, or not‐for‐profit sectors.

## Disclosure

All authors approved the submission to the journal.

This article is derived from the master’s thesis entitled “The effects of music listening and inhaled aromatherapy on pain intensity, anxiety and fear levels in patients undergoing coronary angiography,” conducted at the Institute of Health Sciences, Fırat University. In addition, it was accepted in the scientific research project unit of Fırat University with the project number SYO.22.05.

## Ethics Statement

Before initiating the study, required verbal and written permissions were obtained from patients, the Fırat University Interventional Research Ethics Board, and the institutions where the study was intended to be conducted. Each patient included in the sample was briefly informed on the aim of the study and informed consent was obtained in writing with an informed consent form. During the planning phase for the study, a number was obtained from ClinicalTrials, which is an official institution that reviews and evaluates experimental research and grants approval at the international level.

## Consent

Informed consent was obtained from all individual participants included in the study.

## Conflicts of Interest

The authors declare no conflicts of interest.

## Data Availability

The data that support the findings of this study are available upon request from the corresponding author.
